# Link Between Non-Alcoholic Fatty Liver Disease and Atrial Fibrillation: A Systematic Review and Meta-Analysis

**DOI:** 10.7759/cureus.1142

**Published:** 2017-04-06

**Authors:** Abdul M Minhas, Muhammad Shariq Usman, Muhammad S Khan, Kaneez Fatima, Muhammad A Mangi, Michael A Illovsky

**Affiliations:** 1 GME Internal Medicine, Orange Park Medical Center; 2 Medical Student, Dow Medical College Karachi, Pakistan; 3 Internal Medicine, John H Stroger J. Hospital of Cook County; 4 Civil Hospital, Dow University of Health Sciences (DUHS), Karachi, Pakistan; 5 Internal Medicine, Orange Park Medical Center; 6 First Coast Cardiovascular Institute, Orange Park, Fl, Usa

**Keywords:** atrial fibrillation, liver disease, nafld, meta-analysis

## Abstract

Association between non-alcoholic fatty liver disease (NAFLD) and various cardiovascular diseases has been demonstrated previously. Recent clinical studies have shown that increased circulating levels of γ glutamyl transpeptidase and liver transaminase, markers which are elevated in NAFLD, increase the risk of new-onset atrial fibrillation. We conducted a systematic review and meta-analysis of the available evidence to establish the possible association of increased chances of atrial fibrillation in patients with NAFLD. We extensively searched the PubMed, EMBASE, Cochrane Library, ISI Web of Science and Scopus databases to identify all possible studies that investigated the possible association of NAFLD with atrial fibrillation. Random effect models were used to pool the data between NAFLD and non-NAFLD group. I^2^ testing was done to assess the heterogeneity of the included studies. Our primary outcome was atrial fibrillation. A total of three studies including 1044 patients in the NAFLD arm and 1016 in the placebo arm were included. On pooled analysis, it was observed that patients with NAFLD had 2.5 times significantly higher chance (OR = 2.47, CI = 1.30-4.66, p = 0.005) of developing new-onset atrial fibrillation. Our meta-analysis identifies the paucity of high-quality evidence regarding the association between NAFLD and atrial fibrillation. More studies are needed to confirm the link between NAFLD and atrial fibrillation.

## Introduction and background

Non-alcoholic fatty liver disease (NAFLD) is the leading cause of chronic liver disease in many western countries [1-2]. Approximately 30% of the adult population in these countries are afflicted with NAFLD, and the prevalence further increases to 70-90% in those with obesity or diabetes [3]. In the past, a link between NAFLD and cardiovascular diseases has been demonstrated. Furthermore, recent clinical studies have shown that increased circulating levels of gamma-glutamyl transpeptidase (GGT) and liver transaminase increase the risk of new-onset atrial fibrillation (AF) [4-5]. Both these enzymes are known to be elevated in NAFLD [6]. This suggests that NAFLD could be a predictor of AF.

An association between the two disorders could have important clinical implications for patients with NAFLD, including different treatment approaches, along with an emphasis on prophylaxis against AF. The above information raises an important question: “What are the chances that a patient diagnosed with non-alcoholic fatty liver disease will also develop AF?” Using this question as our hypothesis, we conducted a systematic review and meta-analysis of relevant studies.

## Review

### Methods

Data Sources and Search Strategy

A systemic literature search was conducted using PubMed, EMBASE, Cochrane Library, ISI Web of Science, and Scopus using the search string (non-alcoholic fatty liver disease OR NAFLD OR non-alcoholic steatohepatitis OR non-alcoholic steatosis) AND (atrial fibrillation OR auricular fibrillation OR atrial flutter). Each database was searched from its inception to November 2016. Furthermore, references and citations of each article were manually screened to identify further relevant articles. All the results were transferred to EndNote and duplicate articles were identified and removed.

Inclusion and Exclusion Criteria

Our selection criteria included observational studies investigating the association between NAFLD and AF. Studies which employed well-established criteria for the diagnosis of NAFLD and AF were considered only. Furthermore, only those studies were considered in which the participants did not have a history of myocardial infarction at baseline. Our exclusion criteria included interventional studies, review articles, case reports, case series, book chapters, editorials, and animal studies. Articles dealing with alcoholic fatty liver disease and those concerned with non-atrial arrhythmias were excluded as well.

Data Extraction and Assessment of Study Quality

All articles obtained from the searches were screened by two reviewers, who were blinded to the findings of each other. Documents were selected only if they strictly matched the eligibility criteria. The articles were first shortlisted on the basis of titles and abstracts. Then, full texts were read to assess relevance. Any discrepancies during this process were solved by discussion until a consensus was reached. If a consensus could not be reached, a third reviewer was consulted.

Data from the included studies were extracted onto excel sheets by two independent investigators. Patient characteristics (authors, type of studies, the number of patients, year of publication, age, gender, diagnostic modality of NAFLD, AF assessment, outcome, mean follow-up time, adjustment of covariates, and results) were recorded. The Newcastle-Ottawa scale was used to assess the quality of the studies selected for meta-analysis [7]: a maximum of nine points to each cohort study (four for quality of selection, two for comparability, and three for quality of outcome and adequacy of follow-up) and a maximum score of 10 points to the cross-sectional study (five for quality of selection, two for comparability, and three for quality of outcome). Researches with a score of greater than seven are considered to have a good methodological quality and low bias.

Statistical Analysis

The association between NAFLD and AF was studied using odds ratios (OR). The heterogeneity between studies was approximated by using the Higgins I2 test (I2 <50% was considered acceptable) [8]. The data from component studies were pooled using a random effects model. Furthermore, a forest plot was created to visually assess the OR values and their corresponding 95% confidence intervals across studies. Publication bias was assessed using a contour funnel plot and Begg’s rank correlation test [9].

### Results

Identification of Eligible Studies

In total, 344 potentially relevant abstracts were identified. After duplicates were removed, 63 unique abstracts remained. After examining the full-text publications, 21 publications seemed to meet the inclusion criteria. Of these, 18 were excluded for the following reasons: no available data on the outcome, review articles, alcohol use, non-atrial arrhythmias, and the diagnosis of NAFLD with liver enzymes. Finally, the remaining three studies to existing data met our selection criteria and were included in the meta-analysis.

**Figure 1:**
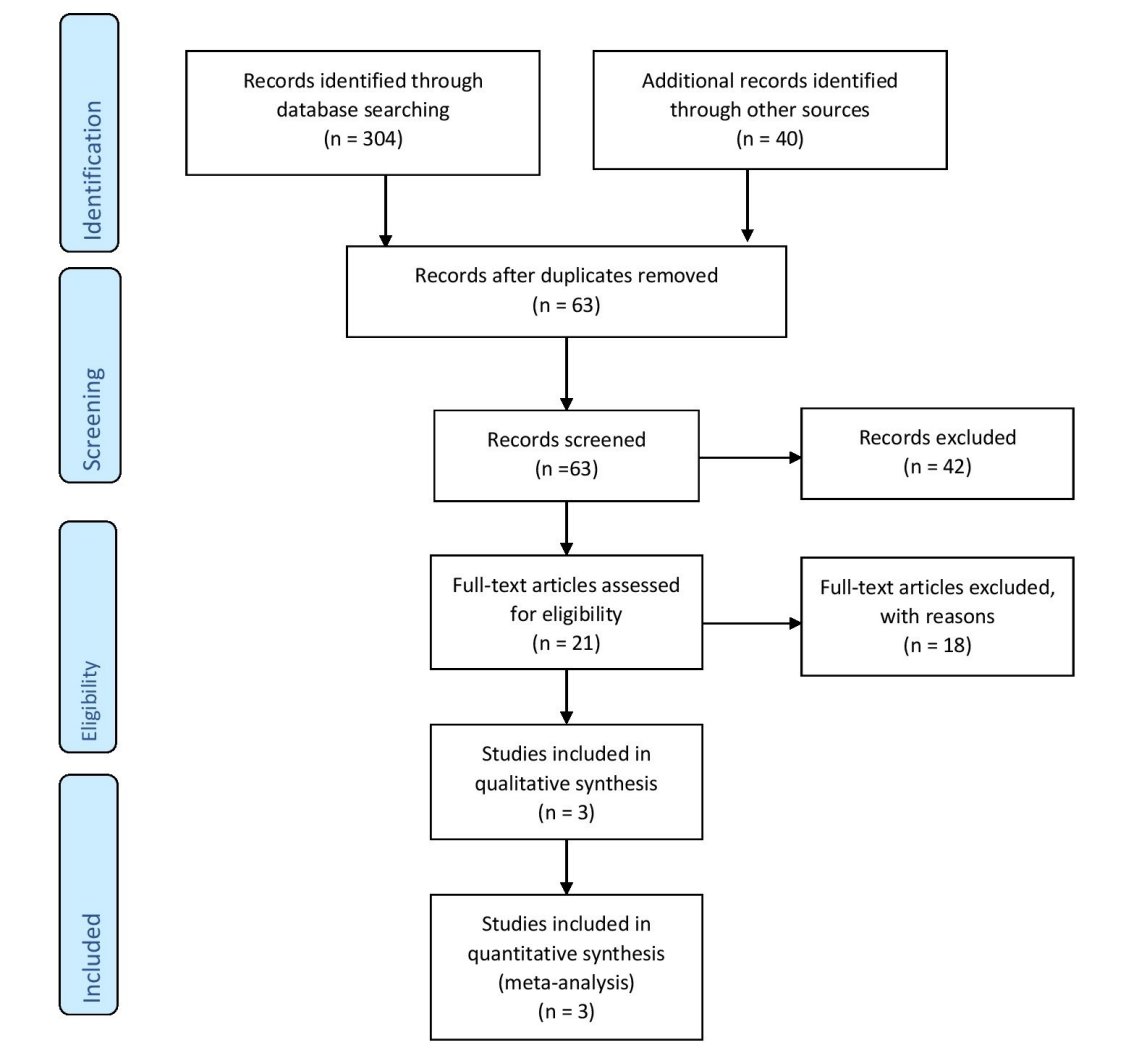
PRISMA flow sheet

The detailed search strategy is outlined using PRISMA flow sheet in Figure 1.

Study Characteristics

Three studies (n = 1044 in NAFLD group and n = 1016 in non-NAFLD group) were selected for meta-analysis [10-12]. The characteristics of these studies are summarized in Table 1. Two of the studies were prospective cohorts [10-11] while the other was a cross-sectional study [12]. All the included studies were published in the previous four years and were single-center studies. The method of assessment of NAFLD and AF was consistent across all studies. All the studies were of high methodological quality. Table 2 and Table 3 show a detailed breakdown of the quality assessment of each study.

**Table 1:** Characteristics of the three included studies ALT: Alanine aminotransferase; ANP: Atrial natriuretic peptide; BMI: Body mass index; BP: Blood pressure; CAD: Coronary artery disease; COR: Crude odds ratio; ECG: Electrocardiogram; HF: Heart failure; hs-CRP: High sensitivity C- reactive protein; IHD: Ischemic heart disease; LVH: Left ventricular hypertrophy; NAFLD: Non-alcoholic fatty liver disease; VHD: Valvular heart disease.

Study	Population	Mean Age (NAFLD/No-NAFLD)	Males % (NAFLD/No-NAFLD)	NAFLD Assessment	AF Assessment	Outcome (AF/Total)	Mean Follow-up Time	Adjustment of Covariates	Result
Karajamaki AJ, 2015 Finland Prospective Cohort Study	Total; 958 NAFLD: 249 Non-NAFLD: 709	52±6/51±6	58/43	Hepatic ultrasonography by a radiologist with 10-year experience	Based on standard 12-lead resting ECG. (noted in medical records)	NAFLD: 37/249 Non-NAFLD: 56/709	16.3 years	Age, sex, diabetes mellitus, BMI, waist circumference, smoking, serum ALT, systolic BP, Left atrial diameter, ANP, CAD and hs-CRP	COR 1.96 (1.29-2.97)
Targher G, 2013 Italy Prospective Cohort Study	Total: 400 NAFLD: 281 Non-NAFLD: 119	63±9/64±9	59.4/57.1	Hepatic ultrasonography performed in all patients by an experienced radiologist, who was blind to the participants' details	Based on a standard 12-lead resting ECG. (noted in routine examination, or in medical records)	NAFLD: 38/281 Non-NAFLD: 4/119	10 years	Age, sex, hypertension, LVH status and PR interval	COR 4.49 (1.6-12.9)
Targher G, 2013 Italy Cross-Sectional Study	Total: 702 NAFLD: 514 Non-NAFLD: 188	65±13/68±14	55.6/49.5	Hepatic ultrasonography was performed in all patients by experienced radiologists, who were blinded to subjects' characteristics	Based on a standard 12-lead ECG done during hospital stay; or from medical history. (noted during hospital stay, or in medical records)	NAFLD: 75/514 Non-NAFLD: 10/188	-	Age, sex, diabetes duration, HbA1c, LVH status, IHD, VHD and HF	COR 3.04 (1.54-6.02)

**Table 2:** Quality assessment of cohort studies *(A), *(B), **(A+B): refer to 'Newcastle Ottawa Quality Assessment Scale for Cohort Studies' [7]

Newcastle Ottawa Quality Assessment Scale for Cohort Studies		
	Karajamaki AJ, et al., 2015	Targher G, et al., 2013
Selection		
1) Representativeness of cohort	*(A)	*(A)
2) Selection of non-exposed cohort	*(A)	*(A)
3) Ascertainment of exposure	*(A)	*(A)
4) Demonstration that outcome of interest was not present at the start of the study	*(A)	*(A)
Comparability		
1) Comparability of cohorts on the basis of design or analysis	**(A+B)	**(A+B)
Outcome		
1) Assessment of outcome	*(B)	*(B)
2) Was follow-up long enough for outcomes to occur?	*(A)	*(A)
3) Adequacy of follow-up cohorts	*(A)	*(A)
Total Score	9/9	9/9

**Table 3:** Quality assessment of the cross-sectional study *(A), -(B), **(A), **(A+B), **(B): refer to 'Modified Newcastle Ottawa Quality Assessment Scale for Cross-Sectional Studies' [7]

Modified Newcastle Ottawa Quality Assessment Scale for Cross-Sectional Studies	
	Targher G, et al., 2013
Selection	
1) Representativeness of sample	*(A)
2) Sample size	-(B)
3) Non-respondents	*(A)
4) Ascertainment of exposure	**(A)
Comparability	
1) Subjects are comparable on the basis of study design or analysis	**(A+B)
Outcome	
1) Assessment of outcome	**(B)
2) Statistical test	*(A)
Total Score	9/10

Meta-Analysis of Primary Outcomes

**Figure 2:**
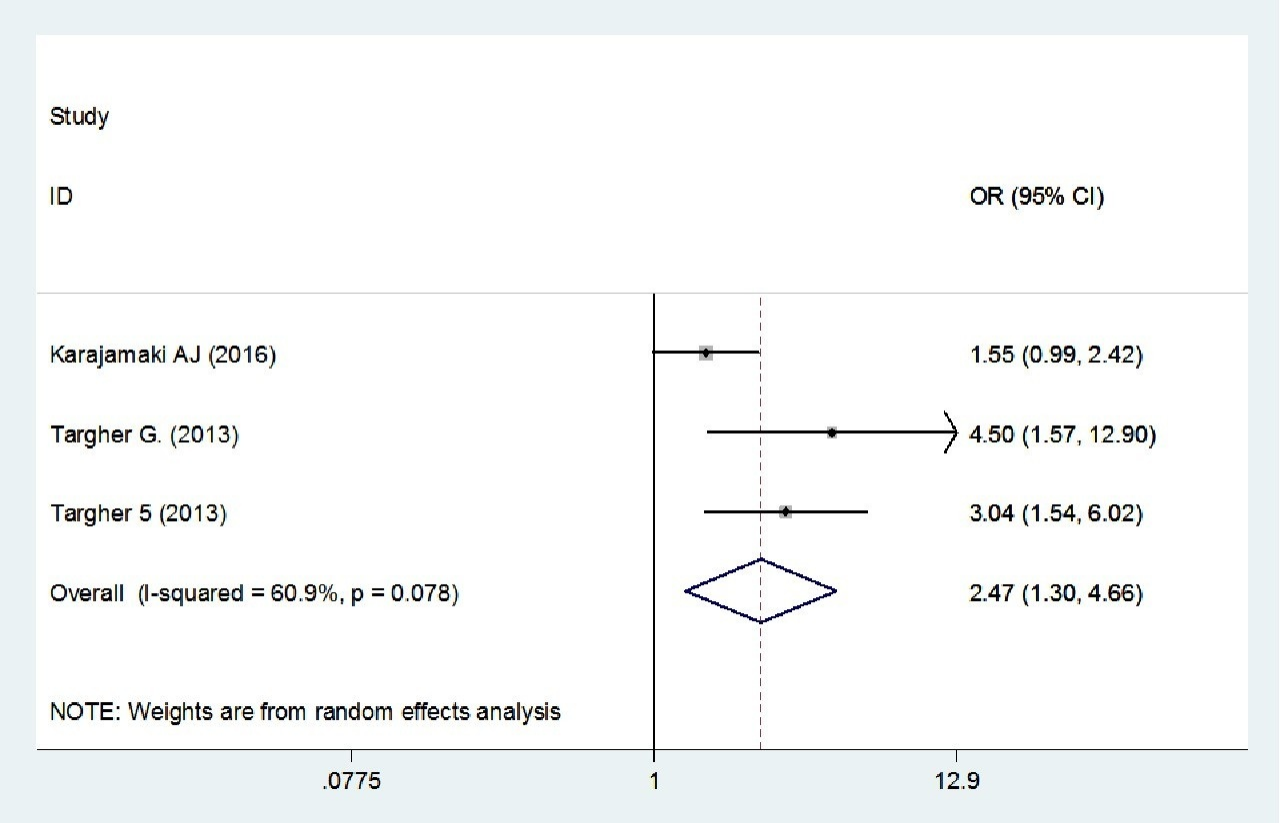
Forest plot summarizing the pooling of studies

**Figure 3:**
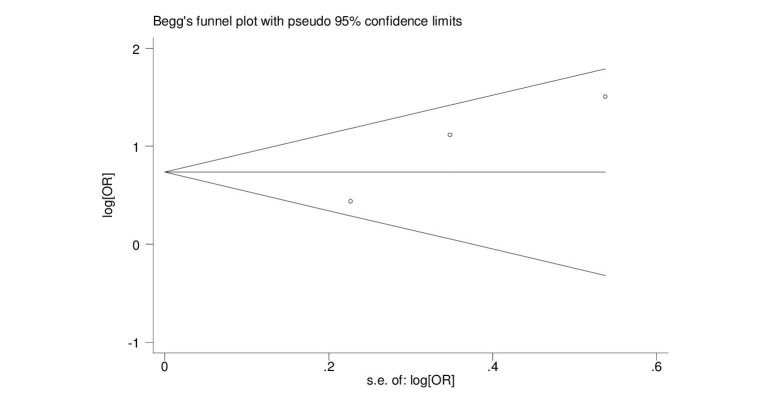
Begg graph for publication bias s.e.: standard error

The pooled ORs showed that patients with NAFLD were around 2.5 times more likely to develop new-onset AF (OR = 2.47, 95% CI = 1.30-4.66, p = 0.005). A forest plot outlining the meta-analysis is shown in Figure 2. We found no evidence of publication bias (Figure 3).

### Discussion

To the best of our knowledge, this is the first systematic review and meta-analysis to study the correlation between NAFLD and AF. Our findings indicate that patients with NAFLD have a statistically significant increased risk of developing AF. The three studies included in our meta-analysis report ORs that, while somewhat variable in magnitude, indicate a positive correlation between NAFLD and AF. Furthermore, in all studies, the association persists and remains statistically significant across a range of subgroup analyses. Although observational studies cannot prove causation, these three studies meet many of the Hill’s criteria for causation [13]. First, the association between NAFLD and AF is statistically significant and most likely not by chance. Second, consistency is seen across the studies – the relationship has been noted in different groups of people and in different locations. Third, a clear temporal association is seen in two of the studies [10-11], where NALFD precedes the onset of AF. Fourth, there is a biological plausibility explaining how NAFLD could lead to AF. All these factors indicate possible causation and set the stage for future research to prove whether NAFLD actually causes AF.

A few hypothetical mechanisms by which NAFLD might lead to AF have been proposed. First, there is evidence that NAFLD independently causes systemic inflammation. The accumulation of fat within hepatocytes increases oxidative stress, which in turn causes the release of pro-inflammatory cytokines [14]. This escalates NAFLD to non-alcoholic steatohepatitis (NASH), and may also lead to systemic inflammation [15-16]. Inflammation is known to induce atrial fibrillation [17-18]. To make matters worse, AF seems to generate and sustain a pro-inflammatory environment, leading to a vicious cycle. Second, NAFLD has been shown to be associated with autonomic dysfunction [19-20]. The irregular sympathovagal stimulation in this condition is associated with an increased risk of atrial fibrillation [21-22] and could be the causal link between NAFLD and AF. Third, NAFLD has been shown to be an independent risk factor for cardiac diastolic dysfunction [23-24], which in turn has been reported to aggravate atrial fibrillation [25-26].

However, the results of our meta-analysis are limited by many factors. The total number of participants in our analysis was only 2,060 which are much less than the amount seen in other meta-analyses [27-28]. Also, the evidence was based on a pooled analysis of observational studies which is limited by the study design and presence of confounders. Furthermore, in two of the studies, all the participants were diabetic – this sample is not a valid indicator of the general population. Another limitation is the fact that included studies did not observe how the risk of AF varied with increasing amounts of fatty accumulation in the liver.

## Conclusions

Our meta-analysis identifies the paucity of high-quality evidence regarding the association between NAFLD and AF. More studies are needed to confirm the link between NAFLD and AF.
